# Adsorption of ^137^Cs on titanium ferrocyanide and transformation of the sorbent to lithium titanate: a new method for long term immobilization of ^137^Cs

**DOI:** 10.1007/s10967-014-3218-7

**Published:** 2014-06-08

**Authors:** Barbara Bartoś, Barbara Filipowicz, Monika Łyczko, Aleksander Bilewicz

**Affiliations:** Institute of Nuclear Chemistry and Technology, Dorodna 16, 03-195 Warsaw, Poland

**Keywords:** ^137^Cs, Nuclear waste, Separation techniques, Ferrocyanide sorbent

## Abstract

Dynamic adsorption of radiocesium on titanium ferrocyanide grains from reactor coolant simulating solution containing salts at moderate concentrations has been investigated. Effective decontamination of the neutral solutions has been achieved, in the amounts of a more than 20 thousand bed volumes. After adsorption the titanium ferrocyanide was transferred to titanates and calcined at 900 °C. The leaching test of the obtained lithium titanates indicates that the loaded adsorbent can serve as an effective primary barrier in nuclear waste repositories.

## Introduction

The fission product radioisotope ^137^Cs causes a big threat to the environment because of its high radiotoxicity and due to the fact that the cesium salts are extremely soluble in water and therefore could migrate in the surroundings. Cesium is chemically similar to sodium, therefore the ingestion of its radioisotopes results in their deposition in the soft tissues all over the body creating an internal hazard, especially to the reproductive system. Therefore, cesium-containing radioactive wastes must be adequately treated prior to their discharge into the environment. Recently, the Fukushima accident illustrated the necessity of developing appropriate processes for the recovery of ^137^Cs from accidental discharge. Existing technologies applied for removing ^137^Cs from nuclear waste streams divided into the solvent extraction, ion exchange, adsorption and precipitation. Solvent extraction is an option for ^137^Cs removal, but this technique has several shortcomings, e.g. extraction coefficients for cesium are relatively small and process needs huge volumes of solvents and stripping agents [1, 2]. Precipitation has been applied in few works, however, the higher pH values typically associated with precipitation methods often result in the precipitation of macro quantities of metals necessitating further separation or decontamination process steps [3].

Column processes utilizing ion exchangers give several advantages over the other techniques. Unfortunately, the proposed ion exchange resins exhibit a low affinity for cesium ions. On the other hand various inorganic ion exchange materials are very selective for cesium and were used for adsorption of ^137^Cs from various liquid wastes [4]. The literature reports ^137^Cs removal from nuclear waste using zirconium and titanium phosphate [5], crystalline silicotitanates [6], ammonium molybdophosphate [7, 8] and various forms of insoluble ferrocyanides, mainly cobalt, zinc, nickel and titanium ferrocyanide [9–11].

The proposed inorganic sorbent must have a high Cs^+^ selectivity in the presence of other cations which are up to many orders of magnitude greater in concentration. The compound must be highly resistant to radiolytic degradation and elevated temperatures resulting from decay heat generation. Finally, the compound after ^137^Cs fixation must be easily transformed to the form allowing final disposal for hundreds of years [12]. However, the proposed sorbents are not suitable as matrices for long-term storage of ^137^Cs. They are decomposed by oxygenation, heat, radiolytic processes and interaction with alkaline conditions of cement materials [13].

One strategy for immobilizing radionuclides is to incorporate them into a ceramic or glass matrix from which they will be difficult to remove [14]. For this purpose ceramics formed by calcinations of titanates has been intensively investigated and it is generally regarded as being more leach resistant than glass [15]. Titanates can be used as a dedicated waste form to dispose off specific wastes or as part of a mixed-phase ceramic assemblage designed to handle wastes of varying composition [16].

Our idea of immobilization of the ^137^Cs radionuclide in the matrix of titanates is based on the initial sorption of ^137^Cs on titanium ferrocyanide (TCF) sorbent, then conversion TCF to the titanates using hydroxide solution and finally calcination of the product to ceramics.

## Experimental

### Synthesis of titanium hexacyanoferrate

Granular samples of titanium hexacyanoferrates were prepared by precipitation from diluted solutions of titanium tetrachloride and potassium cyanoferrate (II) at the mole concentration ratio Ti to Fe(CN)_6_^4−^ equal 10 and pH 1.5. Precipitated TCF were filtered, washed and dried at 70 °C. The dry product was washed again with water, until the supernatant became neutral, then it was dried, ground and sieved. The particles whose diameters were in the range 0.1–0.5 mm were collected for sorption experiments.

### Measurements of distribution coefficients in batch experiments

The distribution coefficient (K_d_) of metal ion is defined as the ratio of the metal ion concentration in the sorbent phase (mmol g^−1^) to that in solution (mmol cm^−3^) at equilibrium. The mass of ion exchanger sample was 50 mg and volume of solution was 10 cm^3^. The solutions were spiked with ^137^Cs. After attaining the distribution equilibrium (48 h of shaking) the aliquots of solution were measured for their radioactivities and compared with those of initial solutions. The K_d_ were calculated according to the equation:

1 $$ {\text{K}}_{\text{d}} = \frac{{ ( {\text{A}}_{\text{i}} - {\text{A}}_{\text{eq}} ) {\text{ V}}}}{{{\text{A}}_{\text{eq}} {\text{ m}}}} $$

where A_i_ and A_eq_ denote the specific radioactivities of the initial solution and at the equilibrium, respectively, V is the volume (cm^3^) of the solution, and m (g) is the mass of the adsorbent.

### Column experiments

The experiments were carried out in a glass column of 4 mm internal diameter. The bed volume of the sorbents was 1 cm^3^, the height was 80 mm. The reactor coolant simulating solution (0.065 M H_3_BO_3_, 2.5 × 10^−4^ M KOH, 2 × 10^−3^M NH_4_OH, carrier free ^137^Cs^+^) was passed through the column. The linear flow rate of the solution was 10 m h^−1^ (125 cm^3^ h^–l^). Due to periodic breaks in the column process, the effective time of sorption was only 20– 30 % of the total time of the experiment. The following parameters of both influent and effluent were continuously recorded: specific radioactivity, pH and conductivity. Samples of effluent were periodically taken out in order to determine the concentrations of Fe(CN)_4_^2−^ anions and the exact values of ^137^Cs radioactivity were measured. The efficiency of radiocesium removal from the solution passed through the sorbent bed under given conditions can be represented by the value of a decontamination factor:

2 $$ {\text{D}}_{\text{f}} = \frac{\text{C }}{{{\text{C}}_{\text{o}} }} $$

where C_o_ and C denote concentrations (or radioactivities) of radiocesium in the influent (the feed solution) and the effluent, respectively. The D_f_ values are determined for given amounts of the effluent, represented by the number of the bed volumes (BV) of the sorbent.

### Calcination and leaching studies

Titanate samples (2 g) loaded with ^137^Cs were calcinated in the furnace at 900 °C for 24 h and treated with water for 35 days. The distilled water was exchanged at increasing time intervals, according to the procedure described in International Standard ISO 6961. All the experiments were carried out at room temperature under reflux apparatus.

## Results and discussion

A great number of transition metal hexacyanoferrates (II) have been studied for their prospective use for selective removal of ^137^Cs from the liquid nuclear wastes. One of the most promising ion exchangers is titanium hexacyanoferrate, which has been used to remove ^137^Cs from the high, medium and low level liquid waste and other radioactive solutions [17, 18]. It is known that in alkaline solutions insoluble ferrocyanide is converted to the corresponding oxides [17]. In the case of TCF in alkaline solutions should be produced titanium oxide or titanates, which should keep the ion exchange properties. To investigate sorption of Cs^+^ on titanates in the presence of competing cations distribution coefficients of ^137^Cs in various 0.1 M hydroxides were determined. Table 1 presents the obtained results.

**Table 1:** Distribution coefficients of ^137^Cs on TiO_2_ in 0.1 M hydroxide solutions (m_TiO2_ = 20 mg, V solution = 10 ml)

Hydroxide (0.1 M)	K_d_ (cm^3^ g^−1^)
LiOH	514
NaOH	225
KOH	37
(CH_3_)_4_NOH	210,000

As shown in Table 1, in alkaline solutions titanates exhibits affinity for Cs^+^ cations. Similarly to the others cations exchangers their selectivity increases with the increase of crystallographic radius and with the decrease of hydrated ionic radius. Therefore smallest influence on ^137^Cs sorption is observed for big hydrated cation of Li^+^ and for dehydrated tetramethylammonium cation.

Before transformation of TCF to metal titanates we tested TCF for adsorption of ^137^Cs on simulated water coolant of PWR reactor in column process.

**Fig. 1:**
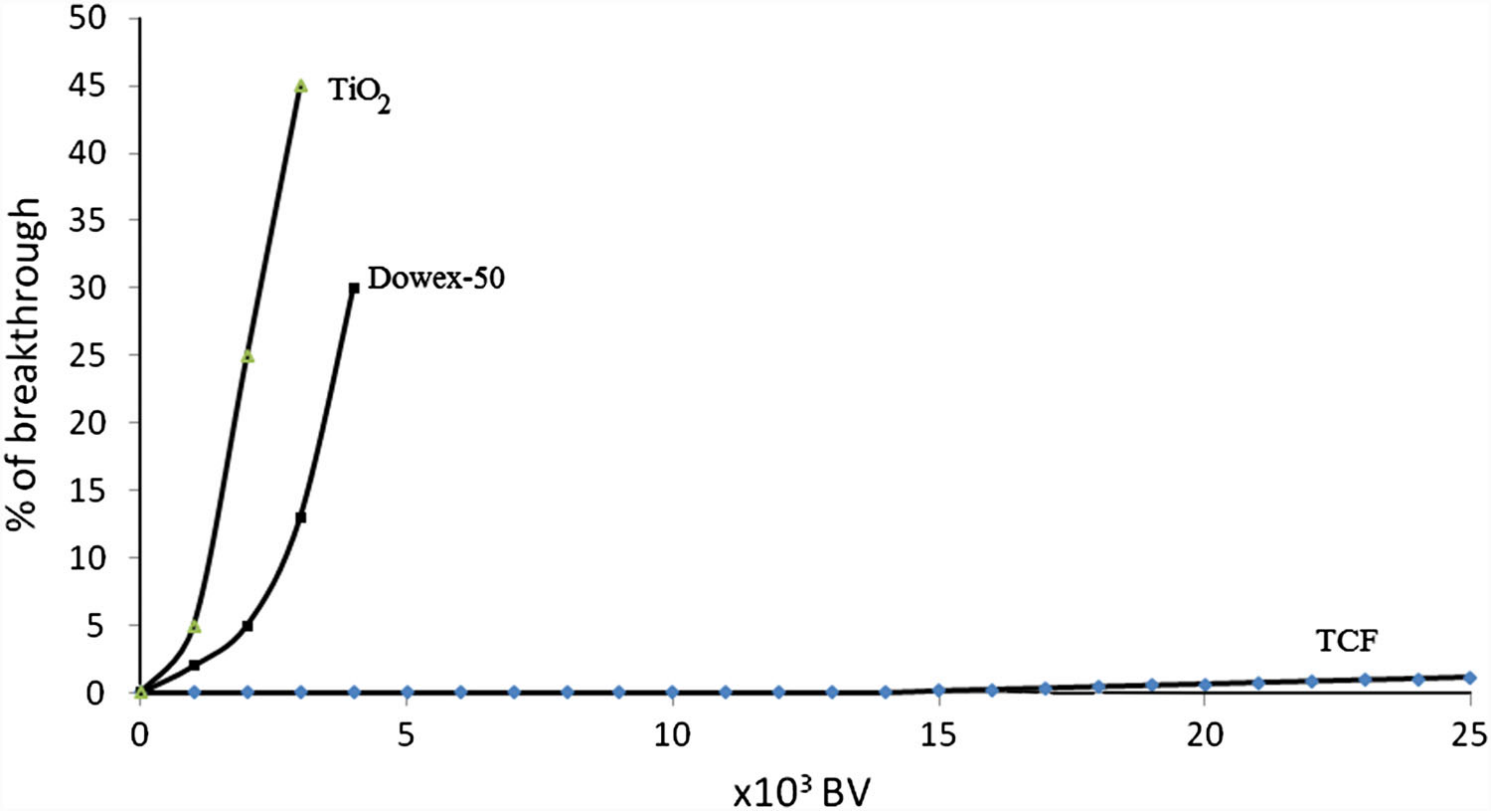
The ^137^Cs breakthrough curves of the TCF column with the solution modeling nuclear reactor coolant

Figure 1 shows the ratios of the specific activity of ^137^Cs in the effluent to that in the influent, plotted against the effluent volume (the number of the bed volumes of the sorbent).

As shown in Fig. 1 in the column experiments we did not observe any breakthrough of the column after passing to 10^4^ bed volumes of the saline solution spiked with ^137^Cs and in the range of 10^4^–2.5 × 10^4^ bed volumes the breakthrough of the column is negligible. In the case of sulphonic resin Dowex 50 and titanium dioxide the breakthrough is observed after passing of less than 10^3^ bed volume. The obtained results indicate that titanium hexacyanoferrate possess selectivity for ^137^Cs similar to other commonly used ferrocyanide sorbents like cobalt, zinc and nickel hexacyanoferrate. In contrast to other ferrocyanide sorbents TCF is unstable in alkaline solutions with a pH higher than 9, while the other ferrocynides are stable in solution up to pH 12. This TCF property allows on his easy transformations to titanium dioxide or metal titanates.

After adsorption of the ^137^Cs on TCF bed, hydroxide solutions were passed through a column and degree of TCF transformation to TiO_2_ and leakage from the column were examined. Hydroxides used were as follows: LiOH, NaOH, KOH, (CH_3_)_4_NOH and C_2_H_5_)_4_NOH. The results are shown in Table 2.

**Table 2:** Percentage washout of ^137^Cs during the transformation TCF to titanates

Hydroxide (0.1 M) solution	Volume of passed hydroxide (bed volumes)	% of ^137^Cs in solution
LiOH	24	0.08
NaOH	24	31
KOH	21	71
(CH_3_)_4_NOH	7	4.7
(C_2_H_5_)_4_NOH	7	2.7

The obtained results indicate that all hydroxide solutions studied convert in 100 % the black TCF to the white TiO_2_ aq or titanates. However, as shown in Tables 1 and 2, when using NaOH and KOH competing influence of the cations K^+^ and Na^+^ causes leakage of ^137^Cs from formed TiO_2_ aq. Much better results have been obtained using tetraalkylammonium hydroxide and particularly lithium hydroxide. This confirms that competitions from big tetraalkylammonium cations and hydrated Li^+^ on sorption of small hydrated Cs^+^ cation is negligible. Similar results were obtained when TCF was used for determination of ^137^Cs radioactivity in reactor coolant [17]. The effective elution of the ^137^Cs from the TCF sorbent was performed by 0.1 M KOH solution, when LiOH eluted only small amounts of ^137^Cs.

Collected samples of the titanates with adsorbed ^137^Cs obtained from transformation of TCF were next calcinated with small addition of zeolite Lucidot and the leaching of radionuclides has been studied. After 35 days leaching test the ^137^Cs activity in the solution was under 10^−2 ^% of the initial sorbent activity.

## Conclusions

Titanium ferrocyanide appears to be an effective adsorbent for radiocesium from neutral aqueous radioactive wastes containing moderate amounts of salts. After transformation to the lithium titanate and calcination the loaded adsorbent can serve as a very effective primary barrier in nuclear waste repositories.
